# *ERG3* and *ERG11* genes are critical for the pathogenesis of *Candida albicans* during the oral mucosal infection

**DOI:** 10.1038/s41368-018-0013-2

**Published:** 2018-03-16

**Authors:** Yujie Zhou, Min Liao, Chengguang Zhu, Yao Hu, Ting Tong, Xian Peng, Mingyun Li, Mingye Feng, Lei Cheng, Biao Ren, Xuedong Zhou

**Affiliations:** State Key Laboratory of Oral Diseases & National Clinical Research Center for Oral Diseases & West China Hospital of Stomatology, Sichuan University, Chengdu 610041, China

## Abstract

The hyphal development of *Candida albicans* (*C. albicans*) has been considered as an essential virulent factor for host cell damage. However, the missing link between hyphae and virulence of *C. albicans* is also been discovered. Here, we identified that the null mutants of *ERG3* and *ERG11*, two key genes in ergosterol biosynthesis pathway, can form typical hyphae but failed to cause the oral mucosal infection in vitro and in vivo for the first time. In particular, the *erg3Δ/Δ* and *erg11Δ/Δ* strains co-cultured with epithelial cells significantly reduced the adhesion, damage, and cytokine (interleukin-1α (IL-1α)) production, whereas the invasion was not affected in vitro. Importantly, they were incapable of extensive hyphal invasion, formation of micro-abscesses, and tongue epithelium damage compared to wild type due to the decrease of the colonization and epithelial infection area in a murine oropharyngeal candidiasis model. The fluconazole (FLC), an antifungal targeted at ergosterol biosynthesis, relieved the epithelial infection of *C. albicans*in vitro and in vivo even under non-growth inhibitory dosage confirming the virulent contribution of ergosterol biosynthesis pathway. The *erg3Δ/Δ* and *erg11Δ/Δ* strains were cleared by macrophages similar to wild type, whereas their virulence factors including agglutinin-like sequence 1 (Als1), secreted aspartyl proteinase 6 (Sap6), and hyphal wall protein-1 (Hwp1) were significantly reduced indicated that the non-toxicity might not result from the change on immune tolerance but the defective virulence. The incapacity of *erg3Δ/Δ* and *erg11Δ/Δ* in epithelial infection highlights the contribution of ergosterol biosynthesis pathway to *C. albicans* pathogenesis and fluconazole can not only eliminate the fungal pathogens but also reduced their virulence even at low dosage.

## Infectious disease: A trigger for fungal toxicity

The damage from oral infection with the fungus Candida albicans can be contained by targeting two cell membrane-building genes. C. albicans cells transition from a rounded shape into long filamentous structures called hyphae prior to invading and damaging host epithelial cells. Researchers led by Lei Cheng at Sichuan University have now identified a key intermediate step between hyphae formation and virulence. They determined that fungal cells lacking either of two genes that manufacture ergosterol, a component of the C. albicans membrane, still form hyphae and attach to epithelial cells. However, these mutant fungi inflict no cellular damage, and did not cause disease in mice. Furthermore, treatment with low-dose fluconazole, a drug that inhibits ergosterol synthesis, rendered the fungus non-virulent without killing it, indicating that this pathway represents an important ‘missing link’ for fungal pathogenesis.

## Introduction

Oral candidiasis, a worldwide medical challenge for fungal superficial infection, is responsible for the high morbidity especially in children, denture wearers and the immunocompromised population, such as human immunodeficiency virus (HIV) infected patients and head/neck cancer patients received radiation or chemo therapy.^1–4^ *Candida albicans* (*C. albicans*) is the most pronounced conditional fungal pathogen colonized in oral cavity.^5^ The filamentous growth of *C. albicans* is considered as the most essential virulence factor for the adhesion and invasion.^6,7^ *C. albicans* can also produce many virulent molecules companied with the hyphal development, such as the cell-surface adhesin and secreted aspartyl proteases (Sap).^8,9^ *Agglutinin-like sequence* (*ALS*) genes encoded cell-surface glycoproteins have important roles in the adherence to host surfaces,^10^ such as agglutinin-like sequence 1 (Als1), which is capable of the inducing adherence to endothelial and epithelial cells.^11,12^ Although the major protein of the hyphal cell wall hyphal wall protein-1 (Hwp1) also functions as a cell-surface adhesion with the ability to mimic mammalian transglutaminase substrates for the formation of covalent cross-linking between *C. albicans* and epithelial cells.^13^ The family of Sap of *C. albicans* is responsible for the adhesion, cell-surface integrity, and tissue damage.^7,14,15^ *SAP6* is the predominant protease gene expressed in the patients with oral candidiasis and the expression occurs concomitantly at the place of tissue damage.^16^

The epithelium is thought to be the first mechanical barrier against tissue invading by *C. albicans*. When the epithelial cells are infected by the *C. albicans* hyphae, they activate the activating protein-1 (AP-1), c-Fos, and mitogen-activated protein kinase 1 (MKP1) to sense the *C. albicans* hyphal damage and produce the epithelial cytokine (such as interleukin(IL)-1α, IL-1β, IL-6, and IL-17), and then recruit immune cells (such as macrophages).^17,18^ However, it remains unclear that which cell components of *C. albicans* hyphae are important for mediating the damage of epithelial cells. Recently, the first fungal cytolytic peptide toxin “Candidalysin” (encoded by *ECE1*) was identified in *C. albicans*.^19^ The *ECE1* deleted mutant can form normal hyphae similar to the wild type strain but not cause the epithelial cell damage, suggesting that candidalysin is a critical factor for the potential of *C. albicans* hyphae to cause invasive mucosal infections and tissue damage without the impact upon filamentous growth. The morphological identity between *ECE1* deletion and wild type strains combined the opposite capabilities on epithelial cell damage highlight the idea that there are “missing links” between hyphal growth and host cell damage. This type of “missing link” genes will provide further insight into the transformation process from commensal to pathogenic state of *C. albicans*, and perhaps additional therapeutic targets.

To combat with *C. albicans* infections, several types of antifungal drugs are developed, such as azoles targeted at ergosterol (key element in cell membrane) biosynthesis,^20^ polyenes binding to ergosterol to form poles in cell membrane,^21^ and echinocandins targeted at cell wall biosynthesis.^22–24^ Fluconazole (FLC), a clinical first-line fungistatic antifungal azole, can bind to Erg11 to inhibit the ergosterol biosynthesis and cause the accumulation of toxic sterols, indicating the importance of ergosterol in *C. albicans*.^25–27^ *ERG3* and *ERG11* are the most important genes in ergosterol biosynthesis pathway and they have key roles in azole drug resistance.^28–30^ However, their contributions to oral epithelial infections are not under investigated. Here we identified that the *ERG3* and *ERG11* genes were also belonged to the “missing link” type of genes for the first time since their deletions were incapable of causing oral mucosal infection similar to *ECE1* gene, but they can also form hyphae. Meanwhile, fluconazole can relieve the epithelial infection even at non-growth inhibitory dosage both in vitro and in vivo, indicating its dual-functional abilities to not only eliminate the *C. albicans* but also inhibit the interaction between fungal pathogens and host cells by reducing the infective virulence.

## Result

### *ERG3* and *ERG11* genes are critical for epithelial cell damage in vitro

**Fig. 1:**
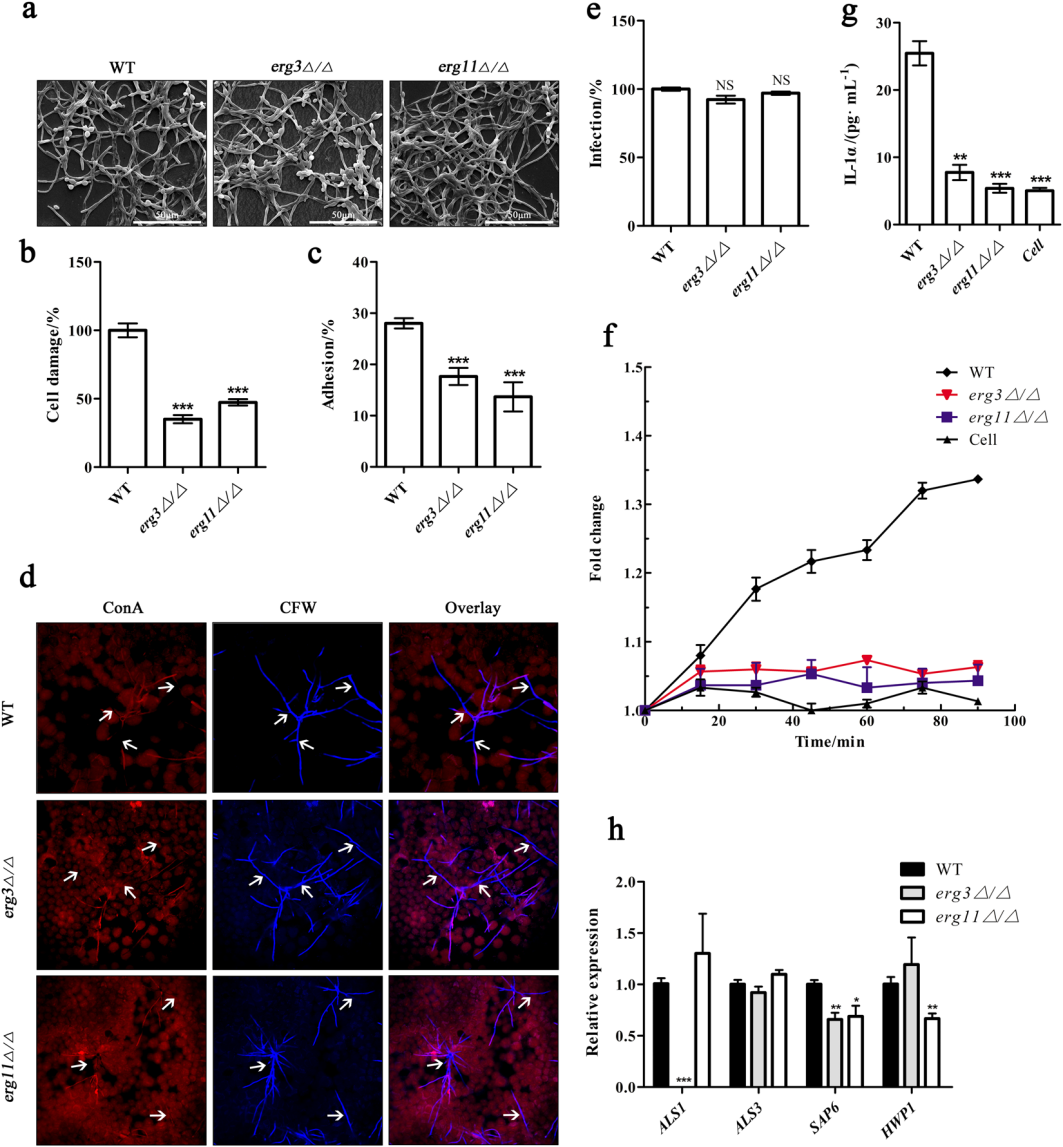
*ERG3* and *ERG11* is required for epithelial cell damage in vitro. **a** Scanning electron micrographs (24 h) showing no obvious difference in hypha formation between *C. albicans* wild type (WT), *erg3Δ/Δ* strain, and *erg11Δ/Δ* strain after infection of TR146 epithelial cells. **b** Cell damage reflected by LDH release after 24 h post infection compared to WT stain (1 × 10^5^ cells per mL). **c** Obviously decreased adhesion to TR146 epithelial cells of *erg3Δ/Δ* strain and *erg11Δ/Δ* strain compared to WT strain after 60 min. **d** Imaging of fluorescence staining of invaded hyphae grown on TR146 epithelial cells. Cell wall chitin are stained with Calcofluor White (CFW, post-permeabilization), whereas carbohydrates are shown by Alexa-Fluor-647-labeled concanavalin A (ConA, pre-permeabilization), which respectively distinguish between invading hyphae and non-invading hyphae A composite image showing CFW and ConA image is presented. White arrows show invading hyphae into epithelial cells. **e** Invasion induced by *erg3Δ/Δ* strain and *erg11Δ/Δ* strain invaded into TR146 epithelial cells has no difference after 24 h versus WT strain. **f** Decreased ROS released by TR146 epithelial cells after infected with *erg3Δ/Δ* and *erg11Δ/Δ* strain compared with WT strain (fold change to 0 min). **g** IL-1α production at 24 h post infection, 1 × 10^4^ cells per mL. **h** Relative expression of virulence factors in *C. albicans erg3Δ/Δ* and *erg11Δ/Δ* strain compared with WT strain measured by RT-qPCR. LDH lactate dehydrogenase

The expression of both *ERG3* and *ERG11* genes were significantly upregulated when *C. albicans* strains co-cultured with epithelial cell, indicating the positive relationship between *ERG3* and *ERG11* and the epithelial pathogenesis (Figure S1a, b). Then we subjected wild type, *erg3Δ/Δ* and *erg11Δ/Δ* to epithelial cell culture to probe the functions of *ERG3* and *ERG11* genes during epithelium infection in vitro. The *erg3Δ/Δ* and *erg11Δ/Δ* strains both can form typical hyphae identical with wild type (Fig. 1a), but they were incapable of inducing epithelial cell damage (Fig. 1b) after co-cultured with epithelial cell for 24 h compared to wild type, indicating that *erg3Δ/Δ* and *erg11Δ/Δ* only formed non-virulent hyphae. Meanwhile, the *erg3Δ/Δ and erg11Δ/Δ* strains significantly reduced the adhesion to the epithelial cells compared to wild type (Fig. 1c). Interestingly, both *erg3Δ/Δ* and *erg11Δ/Δ* strains were capable of extensive epithelial invasion and penetrating through multiple epithelial cells same as the wild type after 24 h co-cultured with epithelial cells, in line with the morphological similarity of hyphae between the mutants and wild type strain (Fig. 1d, e). Although the invasion was not affected, both *erg3Δ/Δ* and *erg11Δ/Δ* strains significantly reduced cell damage and inflammatory through the decrease of the reactive oxygen species (ROS) (Fig. 1f) and cytokine (IL-1α) production (Fig. 1g) in epithelial cells compared to the wild type strain. To identify the reason for the non-virulent hyphae of *erg3Δ/Δ* and *erg11Δ/Δ*, we measured the expressions of some important virulence factors. The expressions of *ALS1* and *SAP6* tested in this study were significantly downregulated in *erg3Δ/Δ* strain as well as the obvious downregulation of *SAP6* and *HWP1* in *erg11Δ/Δ* strain (Fig. 1h), respectively, consistent with their incapability of causing the cell damage and induction of ROS and cytokine, indicating the critical roles of *ERG3* and *ERG11* for epithelial infection in vitro.

### FLC protect the epithelial cells from the damage of *C. albicans* in vitro

**Fig. 2:**
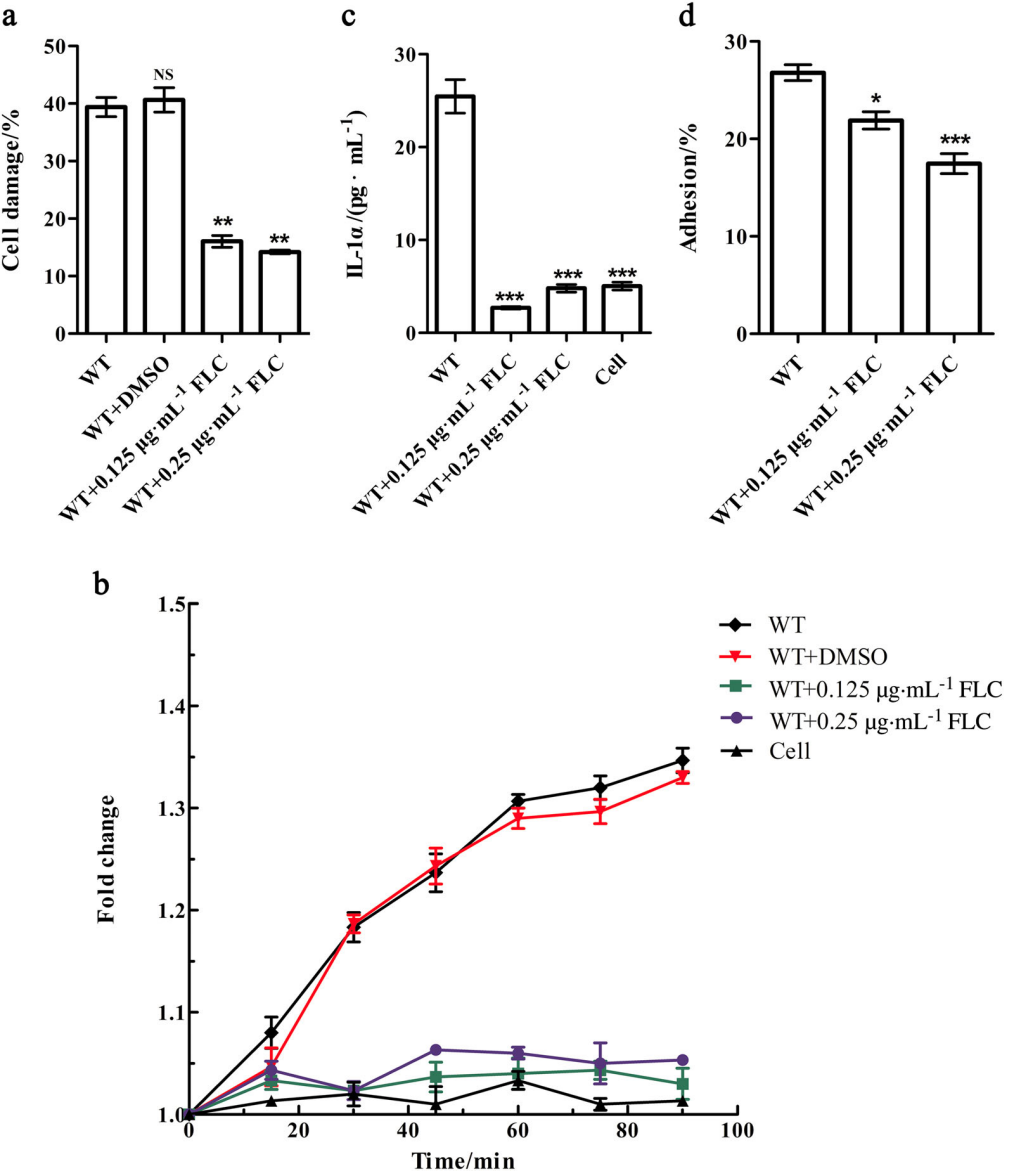
FLC protect the epithelial cells from the damage of *C. albicans* in vitro. **a** Cell damage reflected by LDH release after 24 h post infection compared to wild type (WT) stain without FLC (1 × 10^5^ cells per mL). **b** Reactive oxygen species (ROS) released by TR146 epithelial cells after infected with WT strain with or without FLC (fold change to 0 min). **c** IL-1α production at 24 h post infection, 1 × 10^4^ cells per mL. **d** Obviously decreased adhesion to TR146 epithelial cells by WT strain with FLC after 60 min. FLC fluconazole, LDH lactate dehydrogenase, IL interleukin

As azole drugs can target at the ergosterol biosynthesis pathway,^22^ we employed FLC as an inhibitor to confirm the critical roles of *ERG3* and *ERG11* of *C. albicans* during the epithelial cell infection in vitro. The growth of *C. albicans* was inhibited by FLC at the concentration of 1 µg·mL^−1^ (Figure S2a), so we tried low doses of FLC, at which the growth was not inhibited (Figure S2b), to evaluate its inhibitory ability on the interactions between *C. albicans* and epithelial cell. Interestingly, *C. albicans* wild type stains was significantly reduced the epithelial cell damage at both 0.25 and 0.125 µg·mL^−1^ (Fig. 2a). FLC also significantly suppressed the inductions of ROS and IL-1α productions (Fig. 2b, c). Meanwhile, the non-growth inhibitory concentration could remarkably reduce the adherence rate to the epithelial cells (Fig. 2d). In combination, these results indicate that FLC can not only work as fungal eliminative drug, but also be served as virulence inhibitor when at non-growth dosage due to the critical functions of ergosterol biosynthesis pathway in epithelial cell infection.

### *ERG3* and *ERG11* genes are essential for mucosal pathogenesis in vivo

**Fig. 3:**
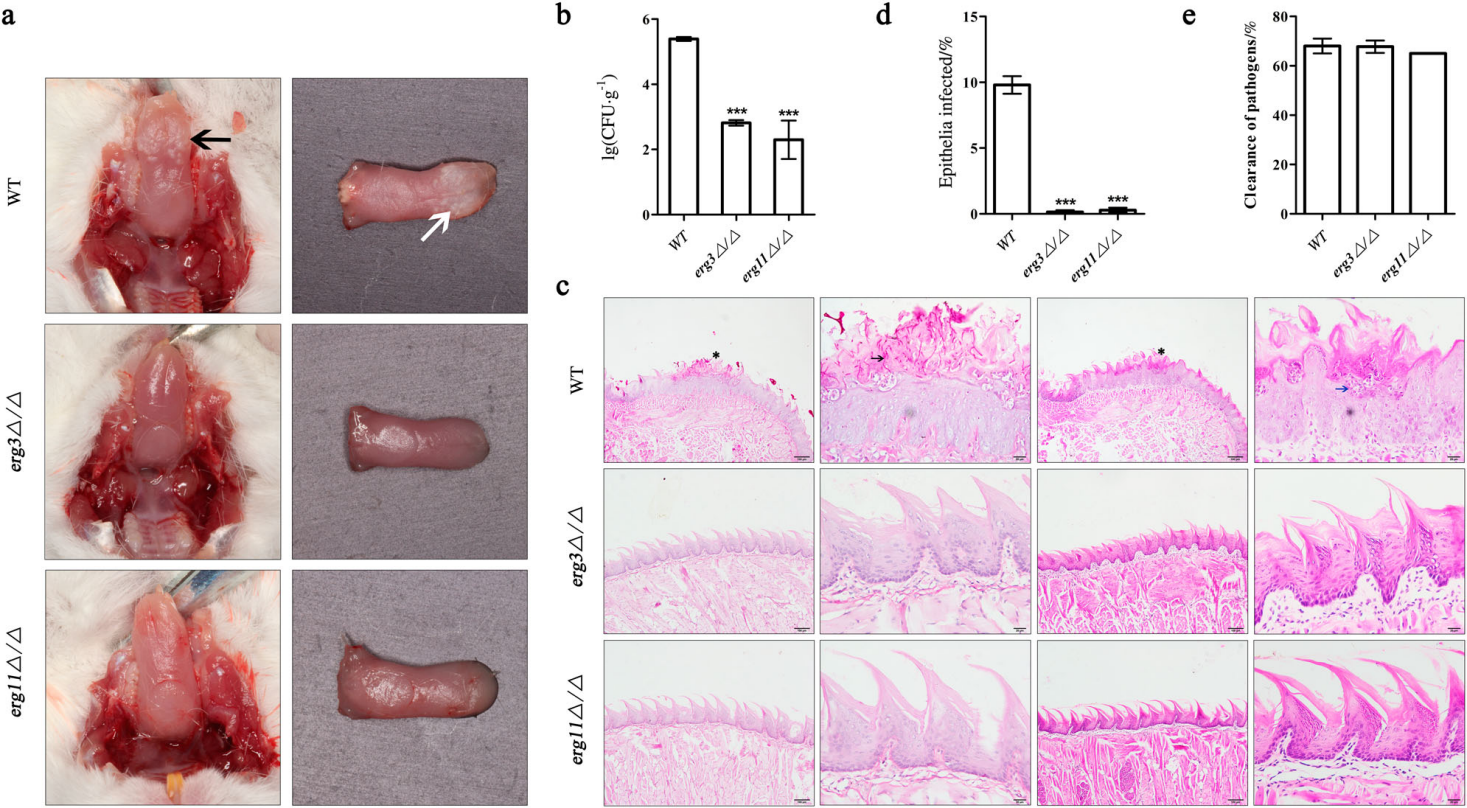
*ERG3* and *ERG11* genes are essential for mucosal pathogenesis in vivo. **a** Images of infected mice tongues with oral candidal leukoplakia after 2-day oropharyngeal infection with wild type (WT), *erg3Δ/Δ*, and *erg11Δ/Δ* strain. Leukoplakia on tongue are indicated in vivo by black arrow, while showed by white arrow on incide tissue. **b** Fungal burdens obtained from the tongues of mice after 2-day oropharyngeal infection with *C. albicans* WT, *erg3Δ/Δ*, and *erg11Δ/Δ* strain. **c** PAS- and HE-stained tongues from mice 2 days post infection by *C. albicans*. Including whole-mount and high-magnification views infected by WT strain, *erg3Δ/Δ*, and *erg11Δ/Δ* strain. Invading hyphae are indicated by black arrowhead and inflammatory cells are showed by blue arrowhead. **d** Average percentage of the mice entire tongue epithelium area infected by WT, *erg3Δ/Δ*, and *erg11Δ/Δ* strain. **e** Susceptibility of *C. albicans* to macrophagocyte have no difference when co-cultured with WT, *erg3Δ/Δ* strain, and *erg11Δ/Δ* strain. PAS Periodic Acid-Schiff, HE hematein eosin

We next assessed the role of *ERG3* and *ERG11* in murine oropharyngeal candidiasis model. The *ERG3* and *ERG11* null mutants were considered as non-virulent hyphae, whereas the wild type served as normal-virulent hyphae control. As expected, the mice infected with wild type strains exhibited typical hyperplastic white plaques on the lingual surface, whereas the *erg3Δ/Δ and erg11Δ/Δ* strains failed to form the lesions (Fig. 3a). Quantification of histology sections including micro-abscesses, the extensive hyphal invasion of the tongue epithelium and tissue damage indicated the typical tongue epithelial infectious disease symptoms when the mice infected with the wild type. In contrast, mice infected with *erg3Δ/Δ* and *erg11Δ/Δ* strains showed no obvious invasive fungal hyphae and no inflammatory infiltrates or tissue damage (Fig. 3b–d). The incapability of *erg3Δ/Δ* and *erg11Δ/Δ* strains to cause mucosal infection in mice was not due to the intolerance to phagocyte challenge as both the null mutants and wild type strains were cleared by macrophage as the same (Fig. 3e), but may mainly related the decrease of virulence factors in *erg3Δ/Δ* and *erg11Δ/Δ* strains (Fig. 1h). Therefore, *ERG3* and *ERG11* genes in *C. albicans* were critical for mucosal infection in vivo.

### FLC can cure the epithelial infection caused by *C. albicans* at low dosage in vivo

**Fig. 4:**
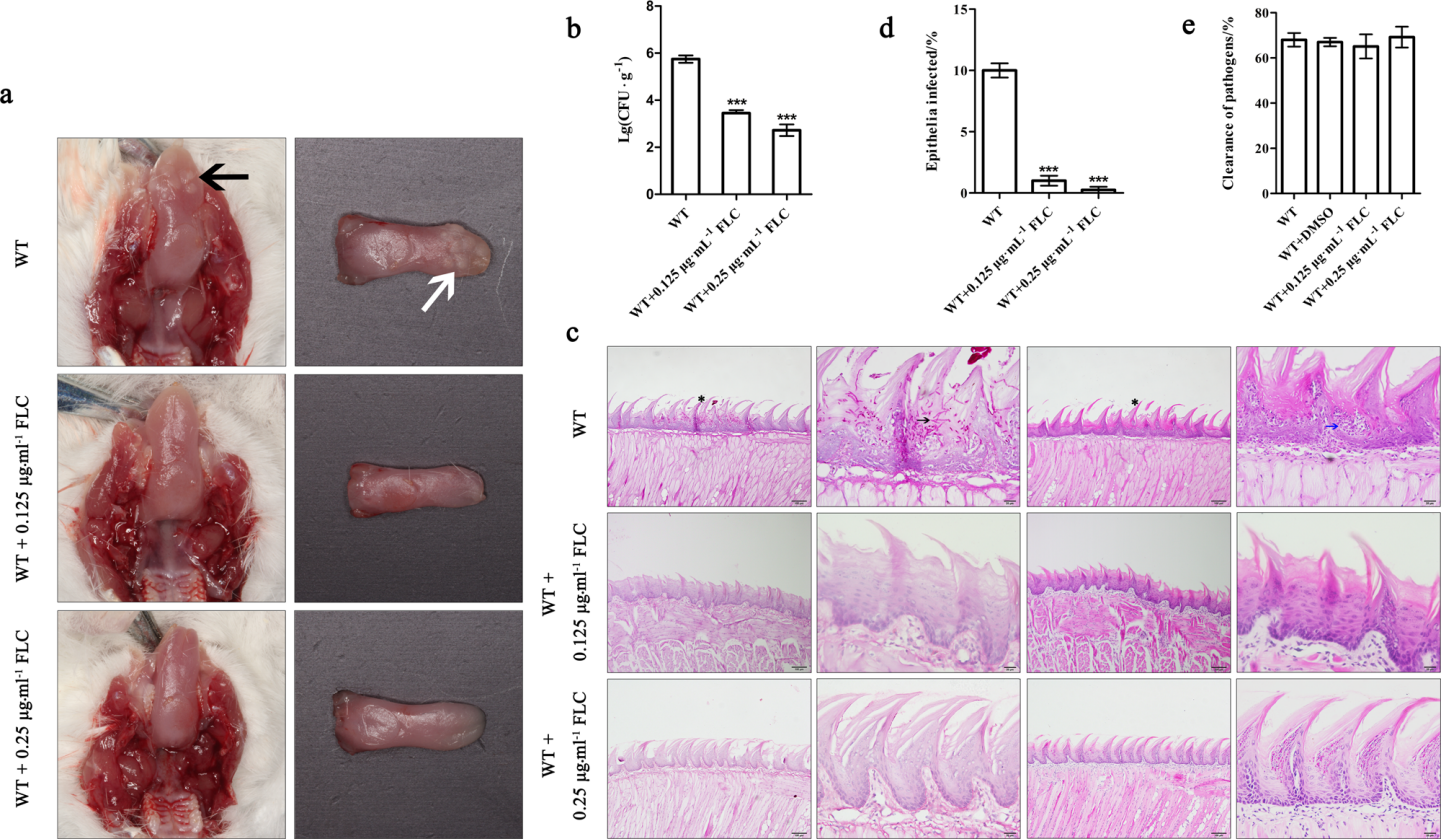
FLC can cure the epithelial infection caused by *C. albicans* at low dosage in vivo. **a** Images of infected mice tongues with oral candidal leukoplakia after 2-day oropharyngeal infection with wild type (WT) strain drinking water with or without different doses of FLC. Leukoplakia on tongue are indicated in vivo by black arrow while showed by white arrow on incided tissue. **b** Fungal burdens obtained from the tongues of mice after 2-day oropharyngeal infection with *C. albicans* WT strain with or without FLC. **c** PAS- and HE-stained tongues from mice 2 days post infection by *C. albicans*. Including whole-mount and high-magnification views infected by WT with or without different doses of FLC. Invading hyphae are indicated by black arrowhead and inflammatory cells are showed by blue arrowhead. **d** Average percentage of the mice entire tongue epithelium area infected by WT strain. **e** Susceptibility of *C. albicans* to macrophagocyte with or without different doses of FLC. FLC fluconazole, PAS Periodic Acid-Schiff, HE hematein eosin

The powerful efficacy of FLC in epithelial cell infection in vitro highlighted the expectation of its potential effects in vivo at low dosage. As expected, after the mice infected by *C. albicans*, the FLC-treated group demonstrated absent white patches and low fungal burdens on the tongues at both 0.25 and 0.125 µg·mL^−1^ compared to the no drug treatment group (Fig. 4a, b). These dosages of FLC also significantly reduced the epithelium infection area, inflammatory infiltrates, and local epithelial damage (Fig. 4c, d), indicating the curative efficacy of FLC even at non-growth inhibitory dosage and suggesting the ability of FLC to block the interactions between fungal pathogens and host. The macrophage clearance rate of *C. albicans* was not effected by FLC (Fig. 4e) in line with the wild type strain, indicated the inhibition of FLC on *C. albicans* virulence.

## Discussion

Ergosterol, the most important component in fungal cell membrane, functions many of the same as cholesterol in animal cells to regulate the fluidity and biogenesis of plasma membrane.^31,32^ Here we identified the functions of ergosterol biosynthesis pathway contributed to the oral epithelial infection caused by *C. albicans* for the first time. The ergosterol biosynthesis dysfunction mutants *erg3Δ/Δ* and *erg11Δ/Δ* failed to damage the oral epithelial cells in vitro and importantly they lost the function to cause the mucosal infection in vivo. Miyazaki et al.^33^ observed that the *ERG3* null mutants showed defective hyphal formation when induced by human serum in vitro and in systemic infected mice. However, when the *erg3Δ/Δ* strain co-cultured with the epithelial cells in vitro, it can form the typical hyphae and show the same invasion rate similar to the wild type strain in our study, indicated that the attachment between *C. albicans* and epithelial cells may regulate the morphological development of *C. albicans*. As the typical hyphae of *erg3Δ/Δ* mutant were observed in vitro, there were no extensive fungal invasion in murine oropharyngeal candidiasis model in line with the observation in systemic *C. albicans* infection mice, indicated the defective virulence of *ERG3* deletion. Becker et al.^34^ found that *ERG3* and *ERG11* were required for virulence in murine model of systemic infection. Our results corroborated the finding that *ERG3* and *ERG11* were critical for the virulence in murine oropharyngeal candidiasis model. Furthermore, we dominated that the reason for that may result from the decrease of key virulence factors such as Als1, Hwp1 and Sap6 in *ERG3* and *ERG11* null mutants instead of their immune intolerance to macrophage. The production of ROS from immune cells, such as macrophage, is an important immune weapon to wipe out *C. albicans* during the early infectious stage. We found that the *ERG3* and *ERG11* gene from *C. albicans* contributed to the ROS production in oral epithelial cells, which may contribute to the *C. albicans*-infected epithelial cell damage. The dysfunction of *ERG11* gene was reported as more sensitive to ROS generated from neutrophils,^35,36^ which may also one of the reasons for the failure of *erg11Δ/Δ* mucosal infection in vivo in our study. In combination of the previous findings in *C. albicans* systemic infection murine model^37,38^ and our results in murine oropharyngeal candidiasis model, ergosterol biosynthesis pathway is proved to be essential for *C. albicans* pathogenesis both in invasive and superficial fungal infection.

The epithelial cells are physically the first defensive surface barrier against *C. albicans* caused invasion and tissue damage. The transition between *C. albicans* yeast and hyphal forms has been proved as the most essential virulence factor by numerous investigations both in vitro and in vivo.^39,40^ However, the recent identified fungal peptide toxin Ece1 functions as the key element to damage the epithelial cell instead of the hyphae of *C. albicans* as the deletion of *ECE1* also formed the morphological identical hyphae compared to the wild type with the same invasive ability.^19^ This type of genes whose deletion will lost epithelial cell damage without the effect on hyphal formation is important to understand the pathogenesis of *C. albicans* during the superficial infection and can be served as new therapeutic potential targets for the treatment of mucosal candidiasis. Our results from the contribution of *ERG3* and *ERG11* in epithelial cell infection model and murine oropharyngeal candidiasis model confirmed that these two genes were another “missing-link” genes between epithelial damage and hyphal development. The *erg3Δ/Δ* and *erg11Δ/Δ* in this study combined previous *ece1Δ/Δ* suggest that this type of “missing-link” genes is likely related to the virulence factors as *ERG3* and *ERG11* deletion decreased the virulence factors in our study while Ece1 acted as toxin to epithelium itself. In view of *C. albicans* infection in vivo, some of its immune evasion-related genes whose deletion will not affect the hyphal development may also lost the infectious ability due to the clearance of immune system in vivo (Y. Zhou et al. unpublished data.2017). Therefore, the “missing-link” genes may typically include virulence and immune evasion correlative genes or pathways, which will be new type of targets for antifungal drug discovery beyond the killing targets. These targets may reduce the fungal drug resistance as the inhibition to these genes will not kill the fungi but cause their incapability in infection.

Owing to the important role of ergosterol in fungal membranes, azole drugs that inhibit ergosterol biosynthesis are widely used for the treatment of fungal infections.^41^ Usually, azole drugs, such as FLC, are clinically used at killing dosage to inhibit the growth of *C. albicans*. Here we identified the multi-functions of FLC, which can not only eliminate the fungi but also inhibit the infective virulence of *C. albicans* both in vitro and in vivo. Especially, when FLC served at non-growth inhibitory dosage, it still reduced the epithelial cell damage in vitro and mucosal infection in vivo indicated its capability to block the pathogenesis of *C. albicans* and suggested that non-growth inhibitory dosage of FLC can also relieve the mucosal infection and be also served as synergistic potentiator to other antifungal compounds for *C. albicans* infection treatment.

## Materials and methods

### Ethics statement

All mouse experiments described in this study were conducted in strict accordance with the guidelines of Ethics Committee of West China Hospital of Sichuan University and the protocols were full approved by this Agency (license number WCHSIRB-D-2016-131). All efforts were made to minimize suffering and ensure the highest ethical and humane standards.

### Chemicals

Fluconazole (98.5%) was commercially obtained from Sigma-Aldrich (China) and dissolved with dimethylsulfoxide (DMSO, Merck-China). It was then stored at −20 °C until use. Concanavalin A-Alexa-Fluor 647 (ConA, Thermo Fisher) were dissolved in sterile phosphate buffer solution (PBS) (10 μg·mL^−1^, stored at −20 °C) and Calcofluor White (CFW, Sigma-Aldrich) (stored at room temperature).

### Strains and media

All the *C. albicans* strains used in this study were listed in Table S1. *C. albicans* strains were maintained on YPD plates (1% yeast extract, 2% peptone, 2% glucose, 2% agar) and then single colony was picked out and subjected into liquid YPD medium at 35 °C overnight. *C. albicans* cells were harvested by centrifugation at 6000 r·min^−1^, 4 °C for 5 min, followed the wash in PBS for three time. The final *C. albicans* suspension was counted by a hemacytometer and then adjusted to the desired concentration in culture medium (Dulbecco’s modified Eagle’s medium (DMEM, HYclone) medium without fetal calf serum).

### Cell lines

Experiments were carried out by using buccal epithelial squamous cell carcinoma line TR146 and R human immortalized macrophage line RAW 264.7 (ATCC, TIB-71™). TR146 was commercially obtained from JENNIO Biological Technology (Guangzhou, China), whereas macrophage RAW cell line was obtained from the American Type Culture Collection (ATCC). These cells were routinely cultured in Dulbecco’s Modified Eagle’s Medium (DMEM, HYclone) supplemented with 10% fetal bovine serum (FBS, Gibco) and 1% penicillin–streptomycin at 37 °C.

### Antifungal susceptibility test and growth measurement

Fluconazole susceptibility measurements were carried out in flat bottom, 96-well microtiter plates (Greiner, Germany), using a broth microdilution protocol modified from the Clinical and Laboratory Standards Institute M-27A methods (National Committee for Clinical Laboratory Standards 2002). Overnight cultures were picked to prepare the strain suspension with medium RPMI 1640 at the concentration of 1 × 10^4^ CFU·mL^−1^. Overall, 2 μL of the fluconazole was added then followed by an additional 80 μL of the strain suspension. The test plates were incubated at 35 °C for 16 h. Minimal inhibition concentrations (MICs) were determined by measuring and comparing the optical densities of the blank control and test wells. Representative aliquot of well-mixed and diluted 100 µL of cultures treated by 0.25 and 0.125 μg·mL^−1^ fluconazole was spotted on YEPD media to monitor cells recovery. All experiments were done in triplicate.

### Relative quantification of differentially expressed genes by real time PCR

*C. albicans* cultures were harvested by centrifugation at 6000 r·min^-1^ at 4 °C for 5 min. The pellets were flash frozen in liquid nitrogen and stored at −80 °C until RNA preparation. RNA isolation was carried out according to the E.Z.N.A.^®^ Yeast RNA Kit (OMEGA Bio-tek.) instructions. Then 1 µg RNA was subjected to the One Step RNA PCR kit (Takara Inc.) to prepare the cDNA according to the manufacturer’s instructions. The RT-PCR were then proceeded by using the SYBR® PremixEx TaqTM kit (Takara Inc.) with following two-step strategy: (1) 94 °C for 30 s; (2) 40 PCR cycles (94 °C for 30 s, a gene-specific annealing temperature for 30 s). All primer sequences are listed in Table S2. Real time PCRs of triplicate samples were performed using CFX 96 Touch^TM^ (Bio-Rad, Hercules, CA, USA). The gene expression level relative to the calibrator was expressed as 2^−ΔΔCT^.

### Cytokine production qualification assay

Cytokine levels produced from cell culture supernatants were determined using Quantikine^®^ ELISA kit (R&D Systems, USA) and a Varioskan Flash machine (Thermo Scientific) according to the manufacture’s introduction. All of the experiments were performed in triplicates.

### Cell damage assay

TR146 cells were grown to confluence on 24-well plates for 48 h in DMEM medium. Lactate dehydrogenase (LDH) array were conducted to determine the TR146 cell damage after the cells co-cultured with *C. albicans* or FLC. Briefly, the culture supernatants were collected after incubation for 24 h and subjected to the LDH activity test by using a Roche cytotoxicity detection kit^plus^ according to the manufacturer’s instructions. All of the experiments were performed at least in triplicates.

### Epithelial cell adhesion assay

TR146 cells were grown to confluence on 24-well plates for 48 h in DMEM medium. After washed by PBS for three times, 1 mL serum-free DMEM of *C. albicans* yeast cells (2 × 10^5^ cells per mL) were added and then followed the incubation for 60 min (37 °C, 5% CO_2_). The non-adherent *C. albicans* cells were aspirated and washed with PBS for three times. The cells were then collected and soaked with sterile double distilled water at 37 °C for 1 h until the epithelial cells were lysed. The suspensions were diluted and spread on YPD plates to derive quantitative candida counts at 35 °C overnight.

### Epithelial invasion assay

TR146 cells were grown to confluence on glass coverslips for 48 h and then infected with *C. albicans* yeast cells (1 × 10^5^ cells per mL) for 24 h in a humidified incubator (37 °C, 5% CO_2_). After the wash for three times with PBS, the cells were fixed overnight (4 °C in 4% paraformaldehyde) and stained with Concanavalin A-Alexa-Fluor 647 (Thermo Fisher) in PBS (10 μg·mL^−1^) for 45 min at room temperature in the dark with gentle shaking to stain the fungal cell wall. After rinsing with PBS, TR146 cells were permeabilized by 0.1% Triton X-100 in PBS for 15 min and fungal cells (invading and non-invading) were stained with Calcofluor White. After rinsing with water, coverslips were visualized using laser scanning confocal microscopy (FV1000, Olympus). The percentage of invading *C. albicans* cells was determined by dividing the number of (partially) internalized cells by the total number of adherent cells. At least 100 fungal cells were counted on each coverslip and all experiments were performed in duplicates on at least three separate occasions.

### Macrophage clearance assay

RAW cells were grown to confluence on 96-well plates for 24 h in DMEM medium. *C. albicans* yeast cells (1 × 10^4^ cells per mL) were added into 100 μL DMEM with serum, incubated for 3 h (37 °C, 5% CO_2_). RAW cells were lysed by soaking with sterile double distilled water in 37 °C for 1 h. The suspensions were diluted and spread on YPD plates to derive quantitative fungal counts.

### Scanning electron microscopy

For scanning electron microscopy (SEM) analysis, TR146 cells were grown to confluence on glass coverslips. After the co-cultured with *C. albicans* (1 × 10^5^ cells per mL) for 24 h, cell media was removed. Post washing with sterile PBS for three times, samples were fixed overnight at 4 °C with 4% paraformaldehyde. Next, samples were dehydrated through a graded ethanol series, and sputter-coated with gold. Samples were then examined and images recorded using a scanning electron microscopy (SEM; FEI, Hillsboro, OR, USA).

### Murine oropharyngeal candidiasis model

Murine oropharyngeal candidiasis model was performed according to the previous description.^19,42^ Briefly, female BALB/c mice were injected subcutaneously with 3 mg per mouse (in 200 μL PBS with 0.5 % Tween 80) of cortisone acetate on days before and post infection. The second day after injected, mice were in a coma for at least 75 min with an intra-peritoneal injection of 5 % chloral hydrate 10 mL·kg^−1^. Then a swab soaked in a 1 × 10^7^ CFU·mL^−1^ of *C. albicans* yeast in sterile saline was placed under the tongue. After 2 days, mice were executed. The tongue was cut out and divided longitudinally in two. After weighed, one half was homogenized and cultured to quantify candida counts on CHROMagar^TM^ Candida plate, whereas the other one was processed for histopathology analysis. To monitor the efficacy of fluconazole, different dosages of fluconazole were added into the drinking water after the mice were infected.

### Immunohistochemistry of murine tissue

*C. albicans*-infected murine tongues were fixed in 10% (v/v) formaldehyde before being embedded and processed in paraffin wax using standard protocols. For each tongue, 5-μm sections were prepared using a Leica microtome and silane-coated slides. Sections were dewaxed using xylene. Then *C. albicans* and infiltrating inflammatory cells were visualized by staining using Periodic Acid-Schiff (PAS) stain and hematein eosin (HE) stain. Sections were then examined by light microscopy. Histological quantification of infection was undertaken by measuring the area of infected epithelium and expressed as a percentage relative to the entire epithelial area.

### ROS assays

TR146 cells were grown to confluence on 96-well plates for 24 h in DMEM medium. The ROS production was determined using a Reactive Oxygen Species Assay Kit (Beyotime, China) according to the manufacturer’s instructions. Briefly, cells were loaded with 10 μmol·L^-1^ H2DCF-DA in serum-free DMEM for 20 min in a humidified incubator (37 °C, 5% CO_2_) in the dark. After washing with serum-free DMEM for three times, 100 mL DMEM medium containing Rosup (100 mg·mL^−1^) was served as positive controls and equal volumes of *C. albicans* strains with or without fluconazole at the indicated concentrations were added. Chemiluminescence was measured at 15 min intervals at 37 °C with a Varioskan Flash machine (Thermo Scientific). Data are expressed as relative luciferase per well TR146 cells over time.

### Statistics

Statistical significance was decided by Student’s *t*-test with Welch’s correction, one-way ANOVA with Dunnett’s or Tukey’s multiple comparison test, or two-way ANOVA with Tukey’s multiple comparison test using GraphPad Prism software. For data plotted on a logarithmic scale the geometric mean is indicated, and data were log-transformed before statistical analysis.

## Electronic supplementary material

Supplementary Figure S1

Supplementary Figure S2

Supplementary Information
