# Supplementary figures and images for: ERG3 and ERG11 genes are critical for the pathogenesis of Candida albicans during the oral mucosal infection
Source: Int J Oral Sci. 2018 Mar 16;10(2):9. doi: 10.1038/s41368-018-0013-2 (PMC5944255; doi:10.1038/s41368-018-0013-2)

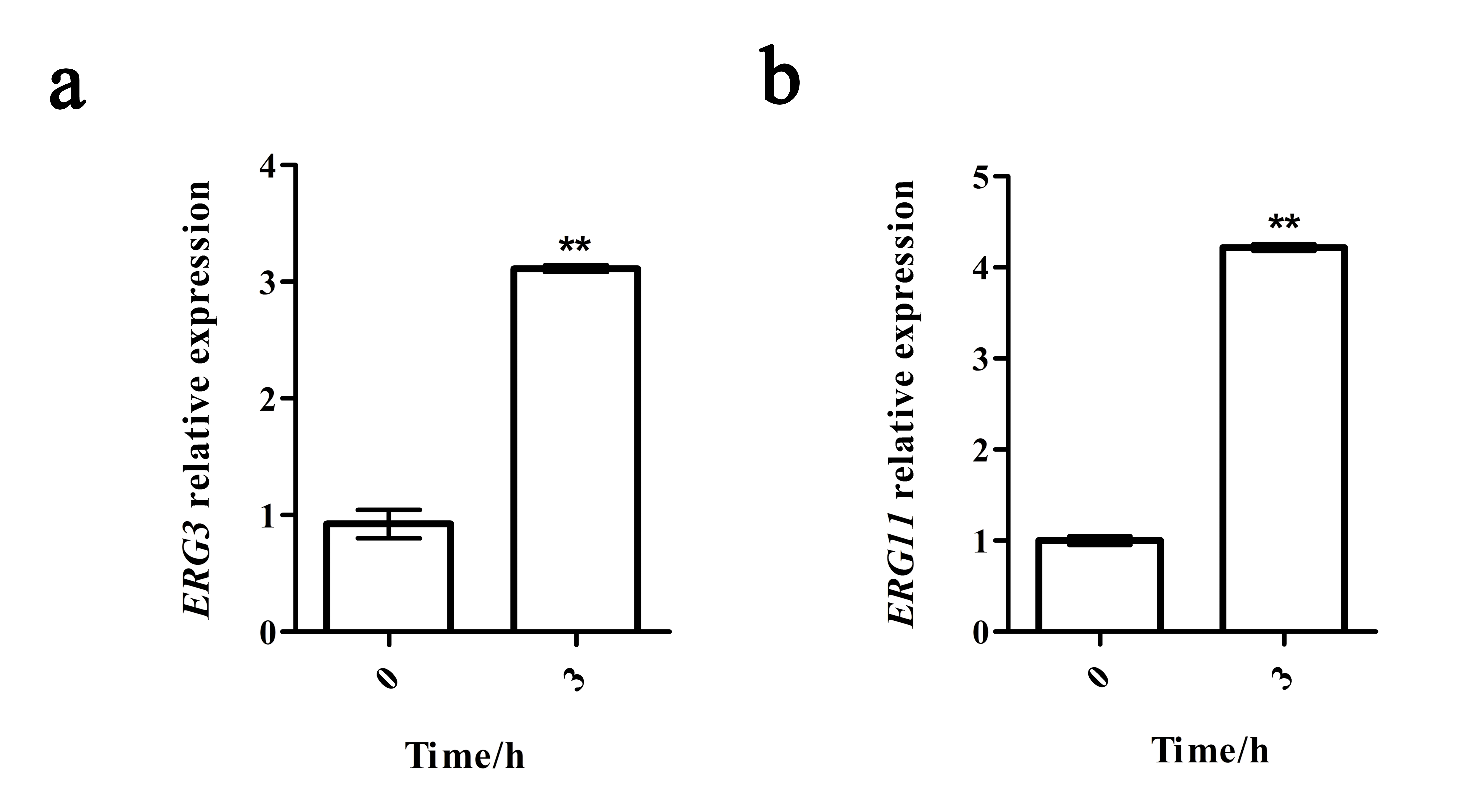

Supplement: Supplementary file 1 — Supplementary Figure S1 [file 41368_2018_13_MOESM1_ESM.tif]

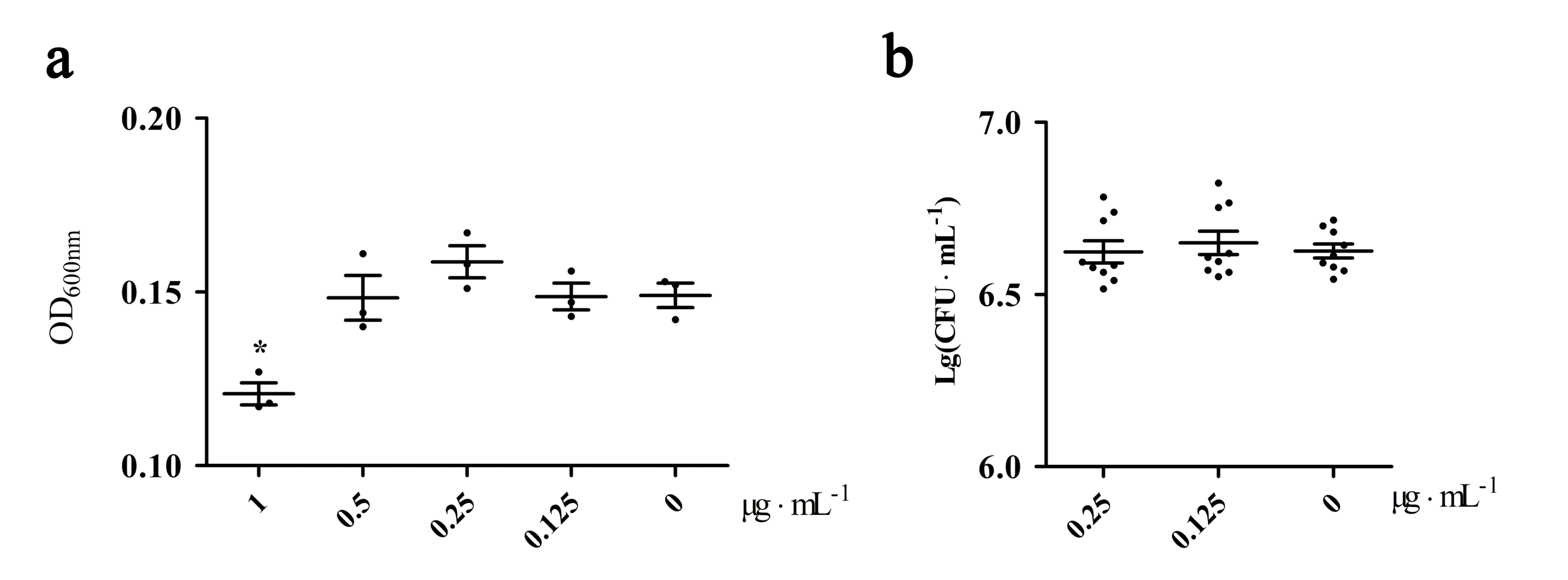

Supplement: Supplementary file 2 — Supplementary Figure S2 [file 41368_2018_13_MOESM2_ESM.tif]
