# Supplementary material for: ERG3 and ERG11 genes are critical for the pathogenesis of Candida albicans during the oral mucosal infection
Source: Int J Oral Sci. 2018 Mar 16;10(2):9. doi: 10.1038/s41368-018-0013-2 (PMC5944255; doi:10.1038/s41368-018-0013-2)
Supplement: Supplementary file 3 — Supplementary Information [file 41368_2018_13_MOESM3_ESM.docx]

**Supplementary**

**Fig S1．*C. albicans ERG3* and *ERG11* expression.**

(a、b) Relative expression (vs *t* = 0) of *ERG3* and *ERG11* in *C. albicans* wild type strain over time after co-cultured with TR146 epithelial cells as measured by RT-qPCR.

**Fig S2. Antifungal activity of FLC.**

1. Optical density of *C. albicans* treated with different concentrations of FLC.
2. Viable cells of *C. albicans* recovered from the treatment of 0.25 and 0.125 µg·mL^-1^ FLC.

**Table S1.***C. albicans* strains used in this study.

| Stain name | genotype | Reference |
| --- | --- | --- |
| Wild type | *C. albicans* CAF2-1: *ERG3/ERG3 ERG11/ERG11* |  |
| *erg11Δ/Δ* | CAF2-1, *erg3A::hisG/ERG3 erg11::hisG/erg11::hisG* | ^30^ |
| *erg3Δ/Δ* | CAF2-1, *erg3A::hisG/erg3B::hisG-URA3-hisG; erg11::hisG/ERG11* | ^30^ |

**Table S2.** Real-time PCR primers used in this study. FW: forward; RV: reverse.

| **Primers** | **Nucelotide Sequence (5’-3’)** |
| --- | --- |
| *18S rRNA* | FW-TCTTTCTTGATTTTGTGGGTGG |
|  | RV-TCGATAGTCCCTCTAAGAAGTG |
| *ERG3* | FW-TGCTTCTCATGCTTTCCATC |
|  | RV-CCATCATGAATCATGACAGTCC |
| *ERG11* | FW-GAGAACGTGGTGATATTGATCC |
|  | RV-GAACCAAGCAGAAGTAGAAGC |
| *ALS1* | FW- CCCAACTTGGAATGCTGTTT |
|  | RV-TTTCAAAGCGTCGTTCACAG |
| *ALS3* | FW-CTGGACCACCAGGAAACACT |
|  | RV-GGTGGAGCGGTGACAGTAGT |
| *SAP6* | FW-AAAATGGCGTGGTGACAGAGGT |
|  | RV-CGTTGGCTTGGAAACCAATACC |
| *HWP1* | FW-TCTACTGCTCCAGCCACTGA |
|  | RV-CCAGCAGGAATTGTTTCCAT |
|  |  |
